# Structural change of retinoic-acid receptor-related orphan receptor induced by binding of inverse-agonist: Molecular dynamics and *ab initio* molecular orbital simulations

**DOI:** 10.1016/j.csbj.2020.06.034

**Published:** 2020-06-25

**Authors:** Shusuke Suzuki, Toshiya Nakamura, Ryosuke Saito, Yuta Terauchi, Kentaro Kawai, Midori Takimoto-Kamimura, Noriyuki Kurita

**Affiliations:** a Department of Computer Science and Engineering, Toyohashi University of Technology, Tempaku-cho, Toyohashi, Aichi 441-8580, Japan; b Faculty of Pharmaceutical Sciences, Setsunan University, 45-1, Nagaotoge-cho, Hirakata, Osaka 573-0101, Japan; c Teijin Institute for Bio-Medical Research, Teijin Pharma Ltd., 4-3-2 Asahigaoka, Hino, Tokyo 191-8512, Japan

**Keywords:** Molecular dynamics simulation, Fragment molecular orbital, Protein ligand interaction, Agonist, Inverse agonist, Nuclear receptor

## Abstract

To elucidate structural changes in the retinoic acid receptor-related orphan receptor gamma (RORγt) induced by the binding of an agonist or an inverse agonist, we conducted molecular dynamics (MD) simulations in explicit water. In addition, *ab initio* fragment molecular orbital calculations were carried out for certain characteristic structures obtained from the MD simulations to reveal important interactions between the amino acid residues of RORγt, and to distinguish the different effects in the binding of an agonist and an inverse agonist on the structure of RORγt. The results elucidate that the hydrogen bond between His479 of helix11 (H11) and Tyr502 of helix12 (H12) is important to keep the H12 conformation in the agonist-bound RORγt. In contrast, in the inverse-agonist-bound RORγt, the side chain of His479 rotates, significantly weakening the interaction between His479 and Tyr502, leading to a conformational change in H12. Therefore, the present molecular simulations clearly indicate that the conformational change in the side chain of His479 in the inverse-agonist-bound RORγt is the main reason for the H12 destabilization induced by the binding of the inverse agonist. Such a conformational change does not occur on the binding of the agonist in RORγt, owing to the strong hydrogen bond between His479 and Tyr502.

## Highlights

• Effects of agonist and inverse-agonist on protein were revealed by MD simulations.
• Structural change in a receptor-related orphan receptor (RORγt) was investigated.
• Binding of inverse-agonist to RORγt causes a rotation of His479 side chain.
• This rotation can be related to destabilizing the helix12 conformation in RORγt.
• Our simulations distinguish the functions of agonist and inverse-agonist on RORγt.

## Graphical abstract

Conformational change of His479 side chain in the inverse-agonist-bound RORγt+3SX; (a) at 228 ns and (b) at 230 ns of MD simulations. By the rotation of His side chain, the interactions between His479 and 3SX/Tyr502 are significantly changed.

**Figure:**
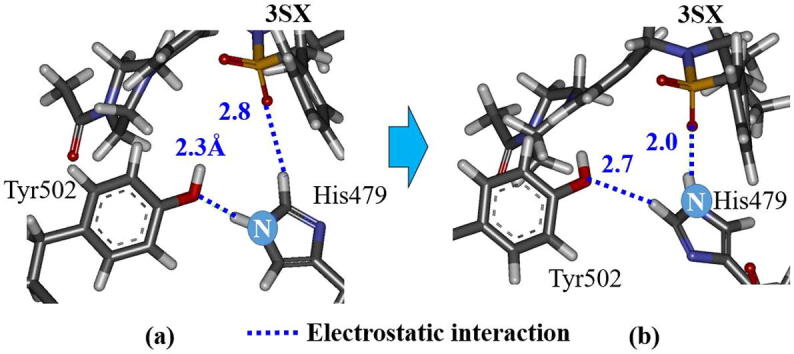

## 1 Introduction

Retinoid-related orphan receptor gamma (RORγ) is a member of the nuclear receptor superfamily. By alternative transcription initiation and splicing of the same gene of RORγ, two isoforms RORγ1 and RORγt are generated. RORγt was found to play an important role in controlling pro-inflammatory gene expression implicated in the pathology of several major autoimmune diseases [1].

RORγt is a transcription factor involved in the production of pro-inflammatory factors [2], [3]. Inflammatory factors function to attack and eliminate pathogens that have entered a body through an inflammatory response. In fact, T-helper 17 (Th17) cells recognize pathogens to produce the pro-inflammatory cytokine, interleukin 17 (IL-17) [4], [5], [6], which produces an inflammatory reaction to the pathogens and removes them. However, excessive secretion of IL-17 causes inflammation in the body (eyes, skin, joints, intestines, etc.), leading to inflammatory diseases, and consequently symptoms such as pain, itching, and fever [7]. By suppressing the activity of RORγt in patients with an inflammatory disease, the excessive secretion of IL-17 can be reduced, leading to a reduction of the inflammation [8]. Accordingly, RORγt has attracted a lot of attention as a target protein for treating inflammatory diseases [9], [10], [11].

Nuclear receptor proteins generally contain several α-helix secondary structures. RORγt has twelve α-helix structures, and helix12 (H12) located at the end of RORγt was shown to activate the transcriptional activity of RORγt by attracting a transcriptional activator to H12 [12]. Therefore, H12 is recognized as the transcriptional activation domain of RORγt and plays an essential role in controlling the DNA transcription mechanism by RORγt. In fact, the conformation of H12 is drastically changed depending on the ligand bound to RORγt. A variety of RORγt agonists, which promote transcription by stabilizing the conformation of H12, have been synthesized as therapeutic small molecules in cancer immunotherapy [13].

On the other hand, an inverse agonist suppresses the transcription by fluctuating the conformation of H12, making it difficult for H12 to attract a transcriptional activator. As a result, when an inverse agonist binds to RORγt, the transcriptional activity of RORγt is significantly reduced. Consequently, in the development of therapeutic agents for inflammatory diseases, candidate compounds, which destabilize the H12 structure and repress the production of IL-17, have also been proposed.

Molecular dynamics (MD) simulations [14] are a very useful tool for elucidating changes in the structure of protein and its ligand-bound structures. To investigate the effect on the stability of the H12 conformation caused by the binding of a ligand to RORγt, atomistic MD simulations have been previously conducted [15]. In this study, the change in conformations of Trp317 residue as well as H12 was simulated for RORγt with an agonist or an inverse agonist. In the agonist-bound RORγt, Trp317 kept a gauche conformation, whereas Trp317 changed to a trans conformation on the binding of an inverse agonist, in the MD simulations. It was also predicted that this change in the conformation of Trp317 is related to the stability of H12 and the reduction of the transcriptional activity of the inverse-agonist-bound RORγt. However, in these MD simulations [15], the RORγt structure without a H12 domain was used, because the H12 conformation of the inverse-agonist-bound RORγt was not yet determined by experiment. Therefore, any changes in the H12 conformation for the inverse-agonist-bound RORγt could not be investigated.

In a recent study [16], potent RORγt agonists were produced by a novel structure-based functionality switching approach from well-optimized RORγt inverse agonists. In addition, their biochemical response to RORγt was validated by exhaustive MD simulations, which indicated a stabilization of the H12 conformation induced by the binding of the agonists.

In the present study, we constructed some initial structures of the inverse-agonist-bound RORγt with H11 and H12 domains, and investigated the structural changes by MD simulations in explicit water. In addition, we used the structure of the agonist-bound RORγt [17] from the protein data bank (PDB) as the initial structure in the MD simulations to simulate structural changes. By comparing the results of the MD simulations for the RORγt complexes, we attempted to distinguish the effects of the agonist and the inverse-agonist bindings on the RORγt structure. Furthermore, to reveal the reason for the structural change in RORγt induced by the ligand binding, we investigated the specific interactions between the RORγt residues and the corresponding ligands at an electronic level, using an *ab initio* fragment molecular orbital (FMO) method. The results elucidate the essential interactions between the RORγt residues for distinguishing the effects of the agonist and the inverse-agonist bindings.

## 2 Details of molecular simulations

### 2.1 Construction of initial structures of RORγt + ligand complexes with H12

**Fig. 1:**
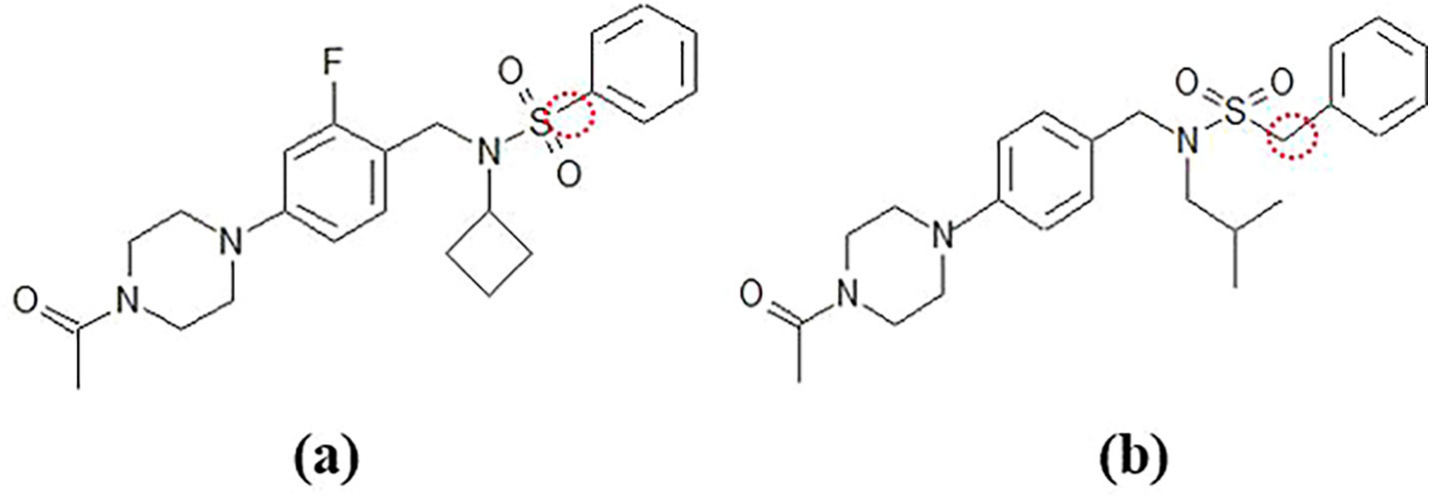
Chemical structures of (a) agonist 3SN and (b) inverse agonist 3SX.

In this study, we employed a RORγt agonist (PDB chemical ID: 3SN) and a RORγt inverse agonist (chemical ID: 3SX). Their chemical structures are shown in Fig. 1. The EC_50_ (effective concentration for 50% activation of the maximum) and IC_50_ (inhibitory concentration for 50% inhibition of the maximum) values of 3SN and 3SX for RORγt are 69 and 47 nM, respectively. Notably, 3SN and 3SX have very similar chemical structures, however, they have the opposite effect on the RORγt activity. The reason for this is not clear at atomic and electronic levels.

The initial structures for the complexes of RORγt with 3SN or 3SX were obtained from PDB, and they were further optimized by classical molecular mechanics (MM) calculations in explicit water. The structure of RORγt with 3SN (RORγt + 3SN) employed is composed from the 264th to the 509th residues of RORγt and contains the H12 domain (PDB ID: 4WPF [17]). On the other hand, in the inverse-agonist-bound RORγt, H12 is not stabilized and fluctuates significantly and as a result the conformation has not been determined by experiment. In the structure for RORγt with 3SX (RORγt + 3SX, PDB ID: 4WQP [17]), the information for the H11′ and H12 domains is therefore missing. To overcome this, we constructed two models for the structure of RORγt + 3SX, as illustrated in Fig. S1 of Supplementary data. In the first model, we replaced the 3SN ligand in the RORγt + 3SN PDB structure with 3SX, owing to the similarities in the chemical structures of the ligands. In the second model, the missing domains (H11′ and H12) were supplemented by the same domains of the RORγt + 3SN PDB structure (4WPF). In the above modeling, a fitting command of the MD simulation program GROMACS [18] was used.

In addition, we optimized the structures of 3SN and 3SX, using the B3LYP/6-31G(d,p) method of the *ab initio* molecular orbital calculation program Gaussian16 (G16) [19]. The charge distributions of the optimized structures were evaluated by restrained electrostatic potential analysis [20], using the HF/6-31G(d) method of G16. Based on the charge distribution, the charge parameters for each atom in the ligand were obtained, and the generalized AMBER force fields (GAFF) [21] for the two ligands in the MM and MD simulation program AMBER12 [22] were constructed.

Protonation states of the His residues in a protein can have a significant effect on specific interactions between the protein and a ligand [23], as such the relevant protonation states were assigned to His residues based on the pKa value predicted by the PROPKA3.1 program [24]. His residues, which possessed a pKa value higher than 6 and were located on the surface of RORγt, were assigned a Hip^+^ protonation, whereas the His residues with a lower pKa value and located inside the RORγt structure were assigned Hie or Hid protonation. The chemical structures of these protonation states are shown in Fig. S2 of Supplementary data. All His residues, except for His453 and His479, were assigned as Hip^+^ protonation. His453 was assigned Hid protonation to reflect the steric hindrance around His453. Notably, His479 has a lower pKa value and no steric hindrance from the surrounding RORγt residues, and therefore His479 can have both Hid and Hie protonation states. In addition, it was confirmed [25] that His479 is important for the stabilization of H12 conformation in RORγt. We therefore considered the both protonation states for His479 and constructed the initial structures of the ligand-bound RORγt structures. These structures were optimized by the MM simulations, and subsequently we determined which structure is more stable based on the total energies evaluated by *ab initio* FMO calculations.

### 2.2 MM and MD simulations for RORγt + ligand complexes in water

The initial structure of the complex was fully optimized in water using AMBER12 [22]. In order to properly consider any solvation effects on the complex, we added a layer of water molecules (8 Å) around the complex. In the MM optimizations, AMBER14SB-ILDN [26], TIP3P model [27] and GAFF [21] were used for RORγt, the water molecules, and the ligand, respectively. The threshold value of energy gradient for convergence in the MM optimization was set as 0.0001 kcal/mol/Å.

Starting with an optimized structure, we carried out 300 ns MD simulations in water and investigated the structural change of RORγt induced by the ligand binding. Particular attention was paid to the changes in structure and conformation of H12. The MD simulations were carried out in a cubic water box, whose size is twice as large as the longest diameter of the complex, and with the complex initially placed at the center of the box. In addition, twelve Cl^−^ ions were added to neutralize the charge of the complex. We placed the Cl^−^ ions near to the side chain of the positively charged Lys and Arg residues located on the surface of RORγt, in order to limit the interactions between the RORγt residues. The MD simulations were executed by setting the periodic boundary condition in the XYZ direction with the water box as a unit, using the MM and MD simulation program package GROMACS Ver.4.5.3 [18].

We first optimized the solvated structure of the complex by the energy minimization method of GROMACS. Subsequently, structural equilibrium calculations were executed by 1 ns MD simulations under constant temperature and pressure conditions (300 K, 1 atm), in order to relax the position and the density of the solvating water molecules. After optimizing the size of the water box, 300 ns MD simulations were conducted under constant temperature (300 K) and volume condition.

### 2.3 *Ab initio* FMO calculations for RORγt + ligand complexes

Finally, we investigated the specific interactions between the RORγt residues and the ligand for certain characteristic structures obtained using MD simulations. The reason for the structural change in RORγt upon the binding of the inverse agonist was investigated using *ab initio* FMO calculations. In the FMO calculations, the target molecule is divided into units, each of which is called “fragment”, and the electronic properties of the target molecule are estimated from the electronic properties of the monomers and the dimers of the fragments [28]. This method can analyze specific interactions between a protein and a ligand involved in the pathogenesis of various diseases with high precision at an electronic level. Accordingly, FMO calculations have been used widely for proposing new ligands for target proteins.

In the present FMO calculations, each RORγt residue, each water molecule and ligand were assigned as separate fragments, because this fragmentation allows us evaluate the interaction energies between the RORγt residues as well as the ligand. The FMO calculation program ABINIT-MP Ver.6.0 [29] was used in the present study. The *ab initio* MP2/6-31G method [30], [31] of FMO was employed to accurately investigate the π–π stacking, NH–π and CH–π interactions as well as the hydrogen-bonding and electrostatic interactions between the RORγt residues and the ligand. In addition, to elucidate which residues contribute to changes in structure, we investigated the inter fragment interaction energies (IFIE) [32] obtained by the FMO calculations.

## 3 Results and discussion

### 3.1 Initial structures of RORγt + ligand complexes

To determine the most stable structures of the RORγt + ligand complexes, we first performed FMO calculations for the MM-optimized structures and evaluated the total energies listed in Table 1. Although the stability of RORγt + 3SN is similar for both the Hid and Hie protonation states of His479, the energy for the Hie protonation is slightly lower than that for Hid. The same trend was obtained for the total energy of RORγt + 3SX. Therefore, we employed the Hie protonation for His479 in both RORγt + 3SN and RORγt + 3SX for the first stage of our study for RORγt. Additionally, the total energies of the two models for RORγt + 3SX are very similar. We employed the second model with the Hie479 protonation, which was produced by supplementing the H11′ and H12 domains, as this model is the most stable among the four structures modeled in the present study. Notably, we will conduct the same MD and FMO simulations in a future study for the structures with the Hid479 protonation, because the total energies are very similar for both the His479 protonations.

**Table 1:** Total energies of RORγt + ligand complexes evaluated by *ab initio* FMO.

Complex	His479 protonation	Total energy (kcal/mol)
RORγt + 3SN	Hid	−103672.17
	Hie	−103672.20
RORγt + 3SX (first model)	Hid	−103613.56
	Hie	−103613.65
RORγt + 3SX (second model)	Hid	−103613.58
	Hie	−103613.66

### 3.2 Structural change of RORγt + ligand during MD simulations

**Fig. 2:**
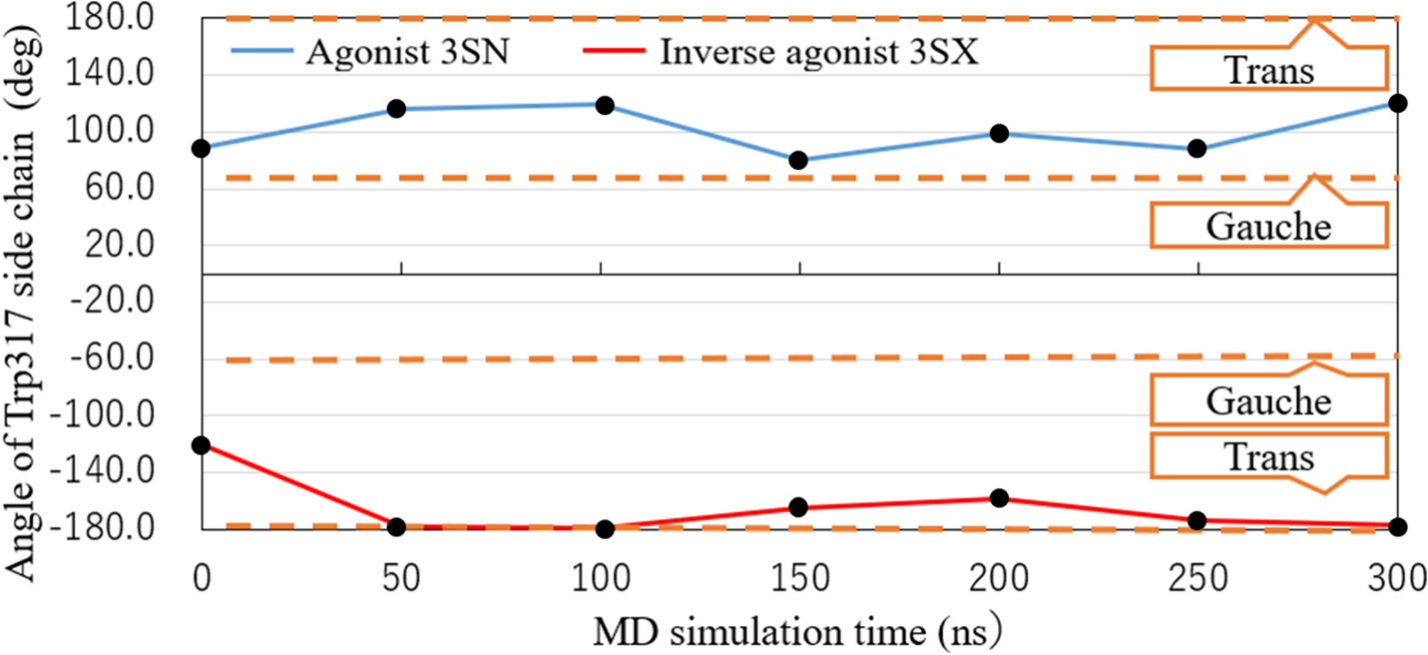
Change in dihedral angle of Trp317 side chain in RORγt + ligand complexes during MD simulations; Gauche and trans conformations have ±60° and ±180° angles, respectively.

To confirm the reliability of our present MD simulations, we investigated the change in conformation of Trp317 in RORγt and compared the results with those obtained by the previous MD simulations [15]. As shown in Fig. 2, the Trp317 side chain has a dihedral angle of 89.2° in the initial structure of RORγt + 3SN, and the angle fluctuates between 80 and 120° during the MD simulations. This result varies from that (60°) of a previous study [15], in which the Trp317 side chain has a *gauche* conformation in RORγt + 3SN. On the other hand, in RORγt + 3SX, the Trp317 side chain has −120° dihedral angle at first and the angle gradually changes into −180°, consistent with the previous result [15] showing that the Trp317 side chain has a *trans* conformation with a 180° dihedral angle.

**Fig. 3:**
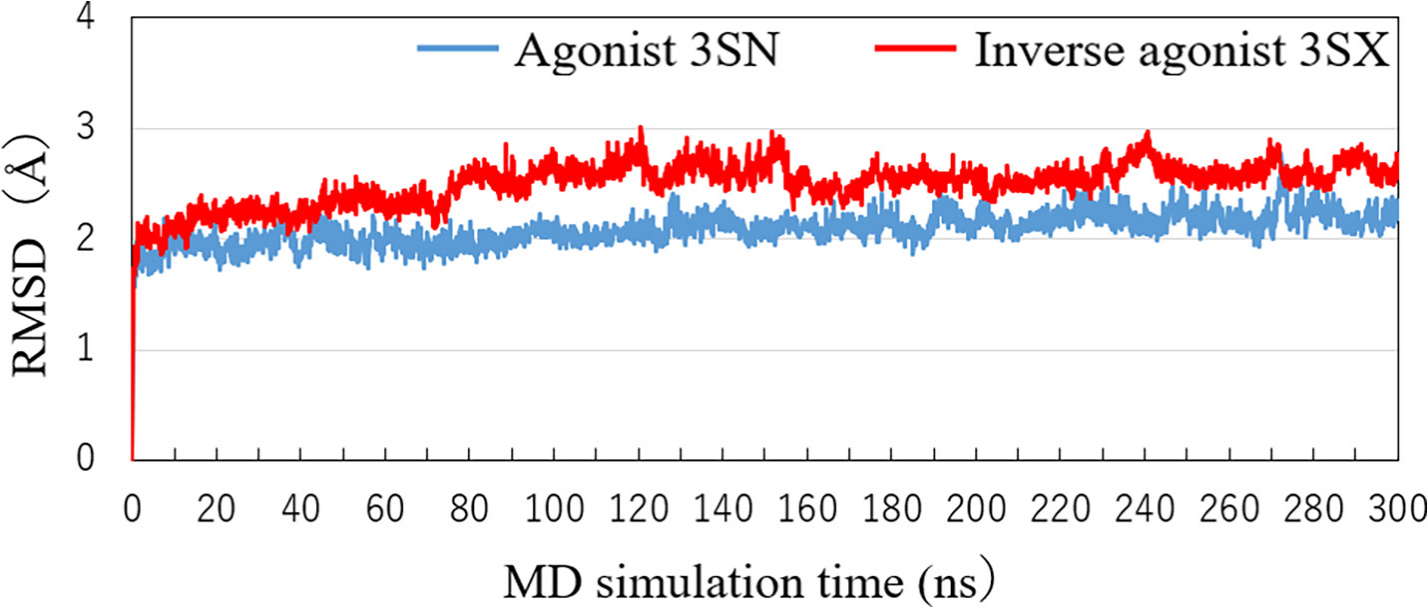
Change in root mean square deviation (RMSD) of a whole structure of RORγt + ligand from its initial structure during MD simulations.

To determine the structural changes in the RORγt + ligand complexes quantitatively, we investigated the root mean square deviation (RMSD) from the initial structure obtained by the MD simulations. As shown in Fig. 3, the RMSDs for both complexes increase rapidly to 2 Å and have no significant change from 10 to 300 ns. Notably, the RMSD for the inverse-agonist-bound RORγt + 3SX is about 0.5 Å larger than that for the agonist-bound RORγt + 3SN. Therefore, the MD results clearly indicate that the inverse agonist, 3SX, induces a more significant change in the RORγt structure.

**Fig. 4:**
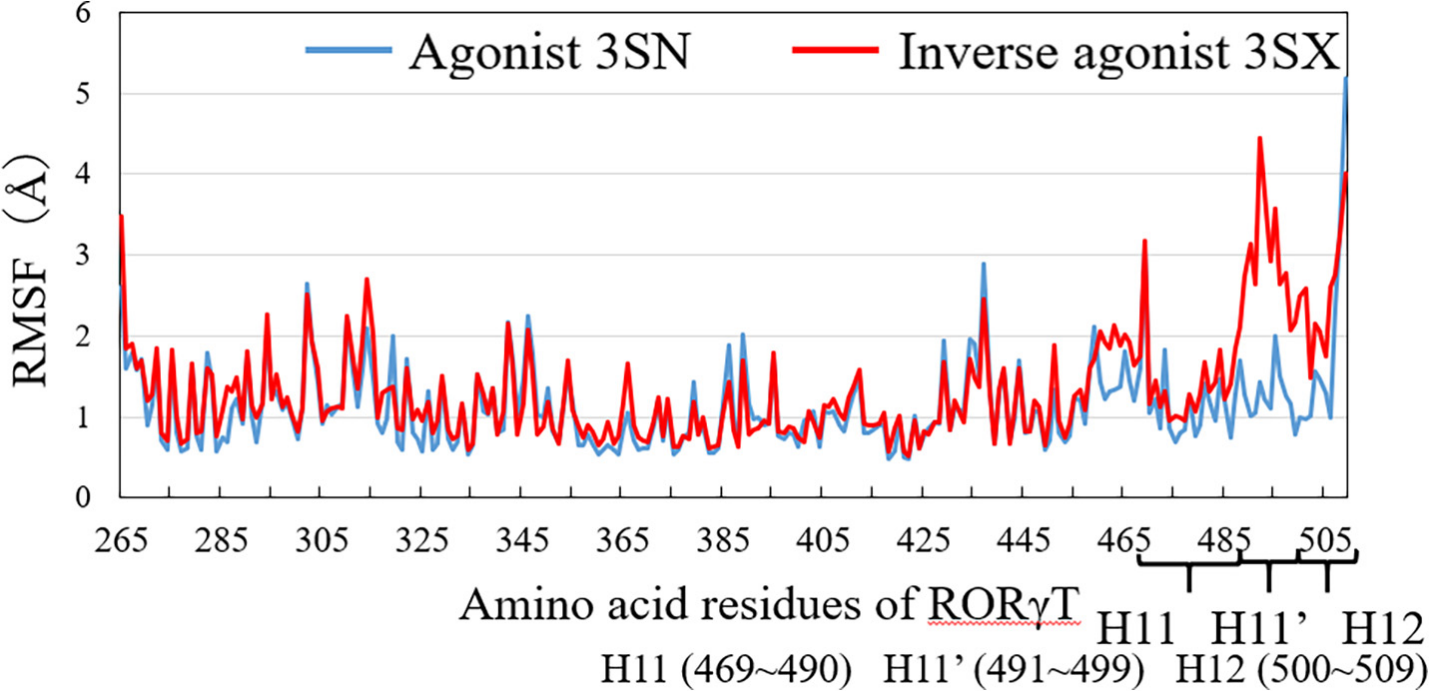
Root mean square fluctuation (RMSF) of each amino acid residue in RORγt investigated by MD simulations.

Moreover, we investigated the root mean square fluctuation (RMSF) for each amino acid residue of RORγt to elucidate which residues fluctuate more significantly in the RORγt + ligand complexes. Fig. 4 specifies that the 469–509th residues of RORγt + 3SX have a larger fluctuation compared to that of RORγt + 3SN. The 469–490th residues make up H11, while H11′ and H12 are composed of the 491–499th and the 500–509th residues, respectively. Accordingly, the present MD simulations indicate that the H11, H11′ and H12 conformations of the inverse-agonist-bound RORγt + 3SX fluctuate more significantly, compared with those of the agonist-bound RORγt + 3SN.

**Fig. 5:**
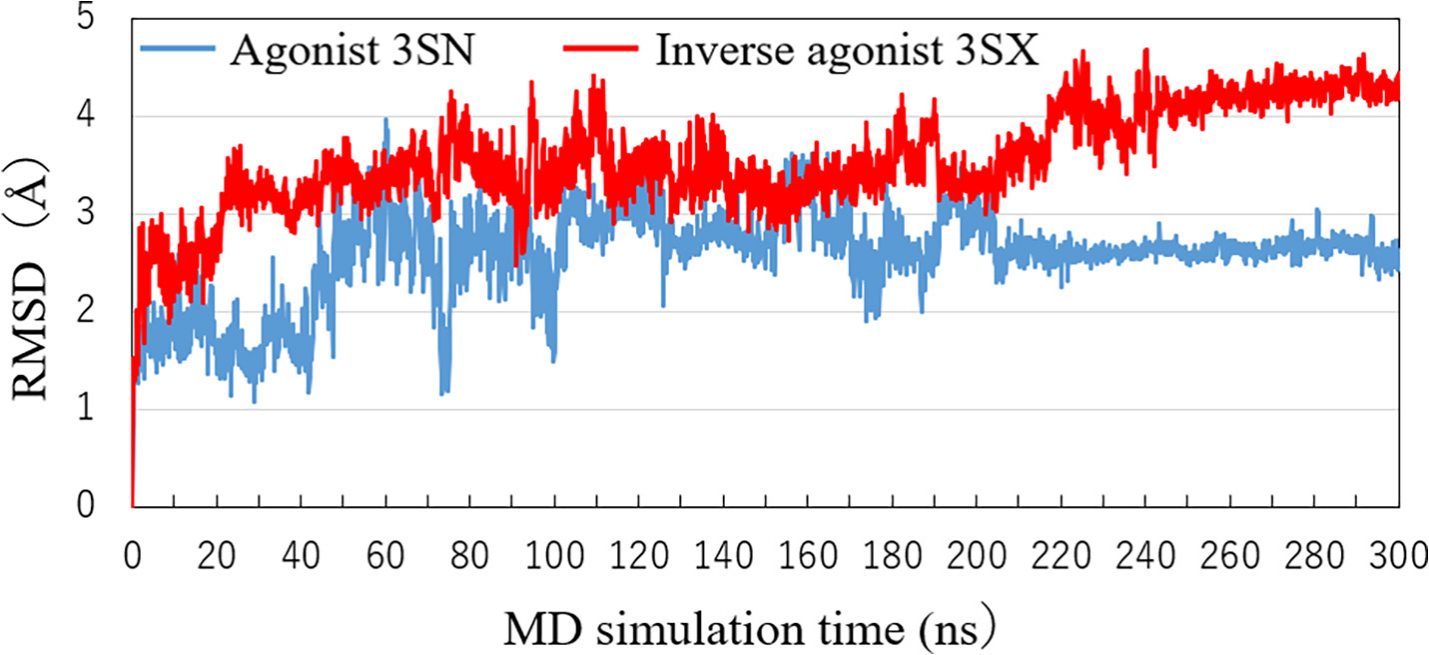
Change in RMSD of H12 in RORγt + ligand during MD simulations.

H12 of RORγt has been recognized as important for attracting a co-activator and stimulating the transcriptional activity of RORγt. We thus investigated the change in the H12 conformation for the RORγt + ligand complexes. As indicated in Fig. 5, the RMSDs for both complexes are significantly different after 200 ns of MD simulations. The RMSD for RORγt + 3SX is considerably larger than for RORγt + 3SN. This result can explain the function of 3SX as an inverse agonist for RORγt. Indeed, at 300 ns of MD simulations, the RMSD for RORγt + 3SX is 1.5 Å larger than for RORγt + 3SN. Therefore, the present MD simulations elucidate that the binding of the inverse agonist 3SX induces a more significant change in the H12 conformation of RORγt.

**Fig. 6:**
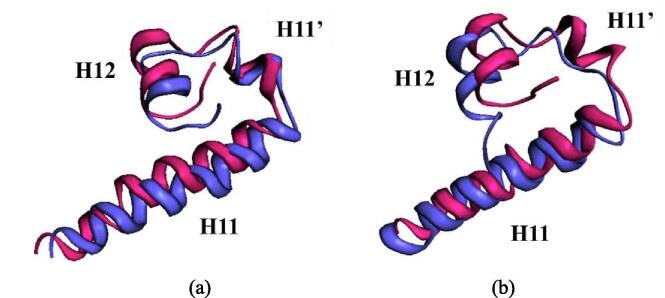
Comparison of structure for H11–H11′–H12 domain in (a) RORγt + 3SN and (b) RORγt + 3SX; red and blue are structures at 0 and 300 ns, respectively. (For interpretation of the references to colour in this figure legend, the reader is referred to the web version of this article.)

To reveal conformational changes in RORγt induced by the ligand, the conformations of H11–H11′–H12 domain at 0 and 300 ns are compared for the RORγt + ligand complexes (Fig. 6). In RORγt + 3SN, the structure of the domain hardly changes during the MD simulations, although the conformation is a little shifted (Fig. 6a). In contrast, H11′ in RORγt + 3SX loses its helical structure (Fig. 6b). As a result, H12 shifts away from H11′. In addition, the conformation of the H12 terminal is significantly changed (Fig. 6b). It is therefore concluded that the binding of the inverse agonist, 3SX, to RORγt induces a significant change in the H11′ and H12 conformations, leading to a large RMSD of the H12 domain induced by the binding of 3SX.

**Fig. 7:**
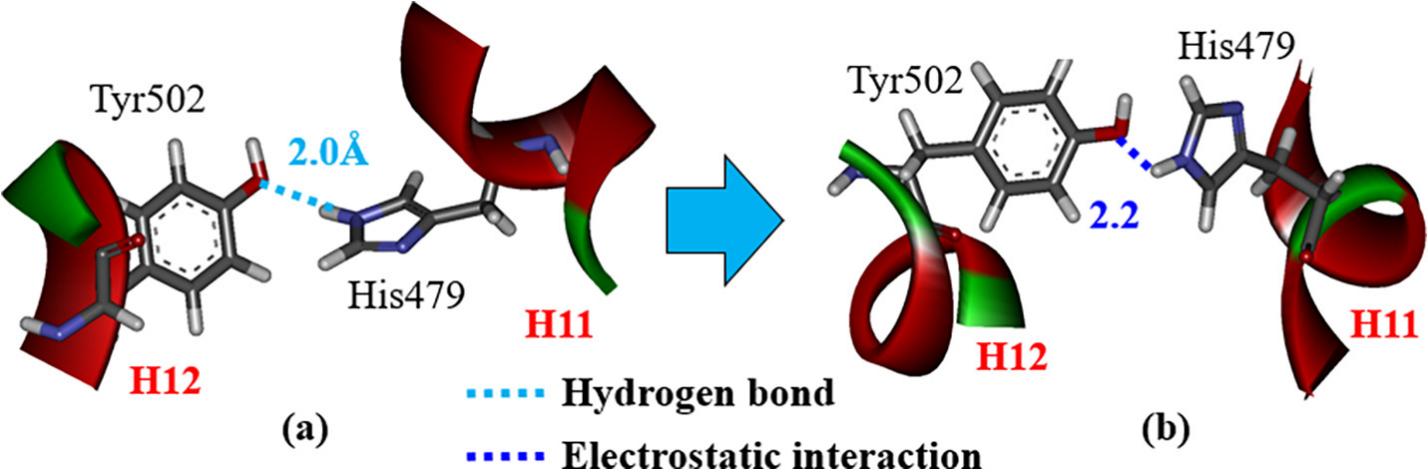
Change in interactions between His479 and Tyr502 in RORγt + 3SN; (a) at 0 ns and (b) at 300 ns.

**Fig. 8:**
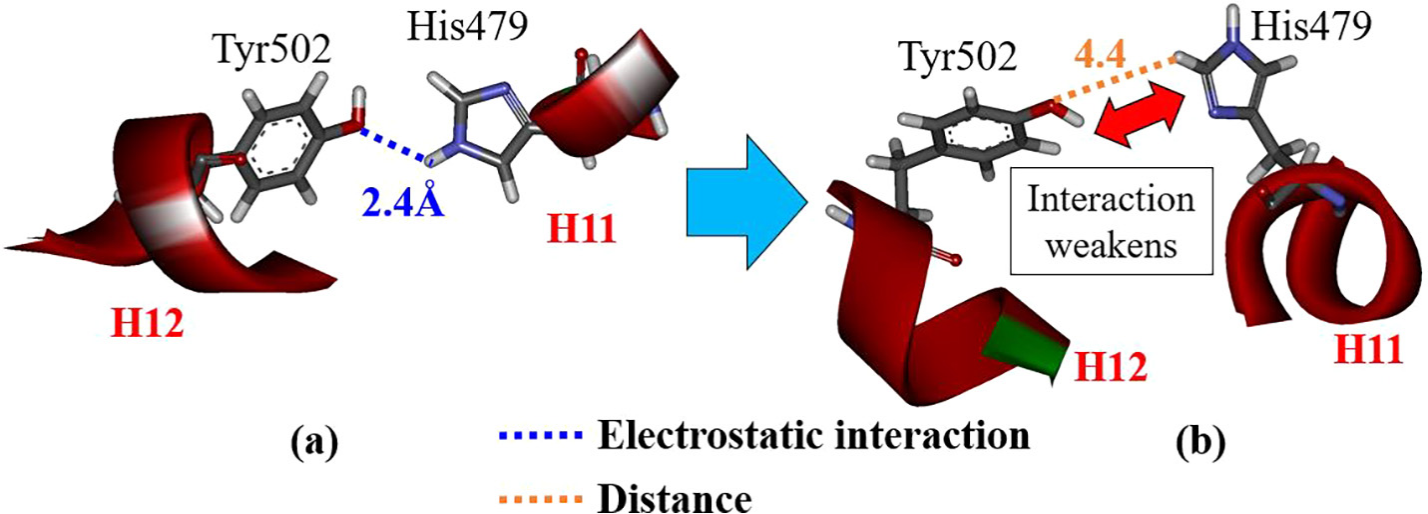
Change in interactions between His479 and Tyr502 in RORγt + 3SX; (a) at 0 ns and (b) at 300 ns.

In the previous MD study [15], the interaction between His479 in H11 and Tyr502 in H12 was confirmed to be important for the stability of H12. In the present study, we investigated the interaction energy between His479 and Tyr502 using *ab initio* FMO calculations, to elucidate the importance of this interaction in the stability of H12. For RORγt + 3SN, the interaction energies for the structures at 0 and 300 ns change by only 1.9 kcal/mol. In fact, the strong hydrogen bond between His479 and Tyr502 at 0 ns is kept even at 300 ns (Fig. 7). By contrast, the interaction energy between His479 and Tyr502 in RORγt + 3SX decreases by 6.6 kcal/mol during the MD simulations. As indicated in Fig. 8, although His479 and Tyr502 strongly interact at 0 ns, they are separated by 4.4 Å at 300 ns. As mentioned above, the present MD and FMO simulations elucidate that the interaction between His479 and Tyr502 is weakened by the binding of 3SX, resulting in the significant change in the H12 conformation. To confirm the reliability of this finding, we will conduct the same simulations for the RORγt + ligand structures with the different His479 protonation.

### 3.3 Change in interactions between inverse agonist 3SX and RORγt residues

**Fig. 9:**
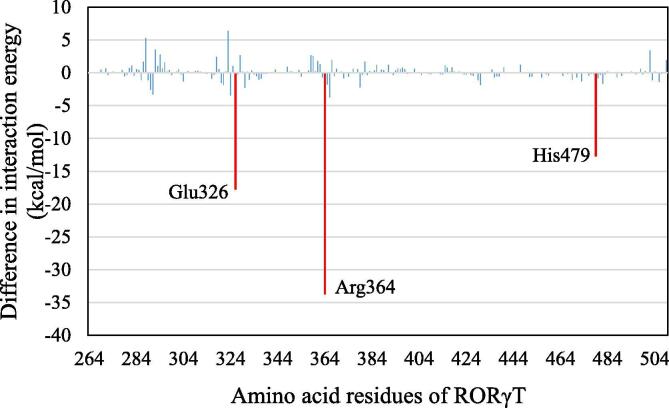
Difference in interaction energies between 3SX and each RORγt residue for the structures at 0 and 300 ns; red-marked bars indicate the residues with difference in interactions larger than 10 kcal/mol. (For interpretation of the references to colour in this figure legend, the reader is referred to the web version of this article.)

To elucidate the reason why only inverse-agonist binding to RORγt induces a significant change in the RORγt structure, we first investigated the change in interaction energies between RORγt residues and the inverse agonist, 3SX. For the structures at 0 and 300 ns, the interaction energies were evaluated by *ab initio* FMO method, and the changes in the energies are shown in Fig. 9. The attractive interactions between 3SX and Glu326, Arg364 and His479 of RORγt were found to be stronger at 300 ns. In particular, the attractive interaction energy between 3SX and Arg364 increased by 33.7 kcal/mol.

**Fig. 10:**
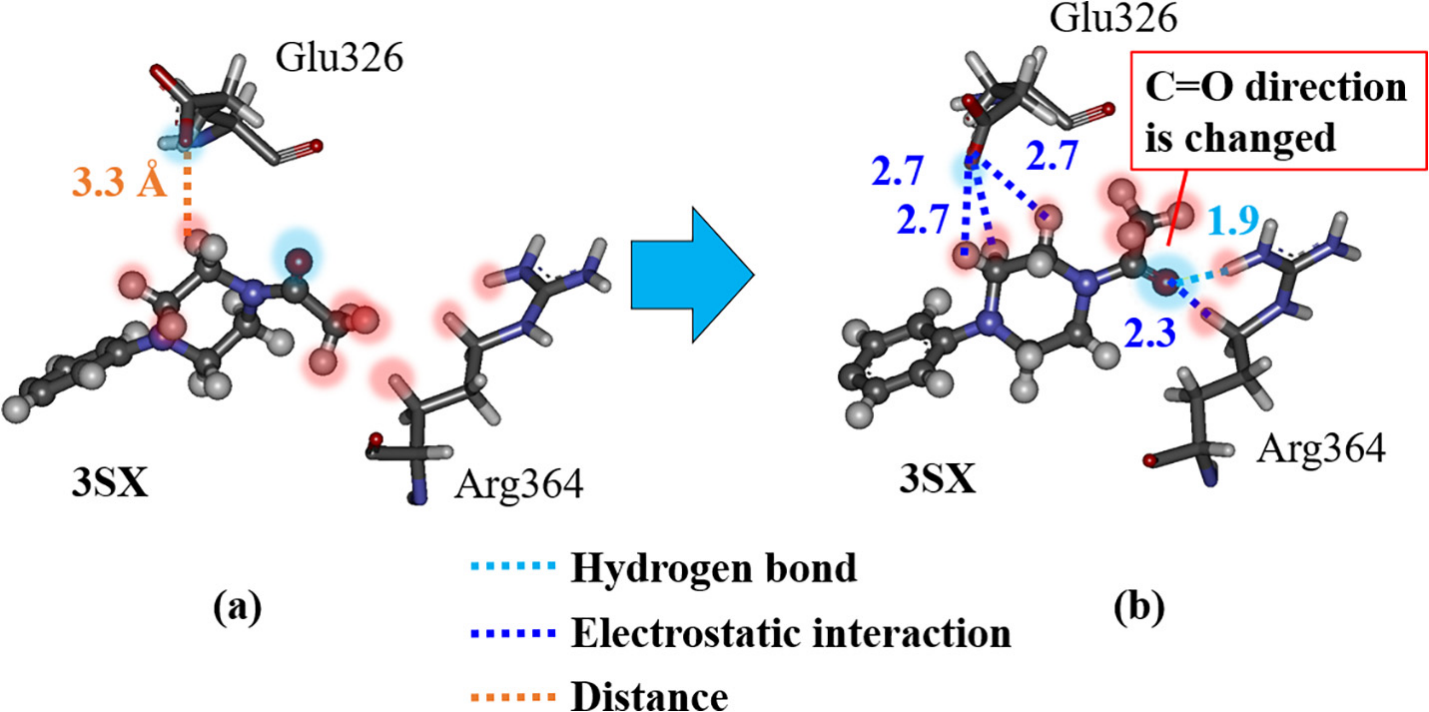
Change in interacting structure between 3SX and Glu326/Arg364 residues of RORγt; (a) at 0 ns and (b) at 300 ns. Red-shaded and blue-shaded atoms have positive and negative charges, respectively. (For interpretation of the references to colour in this figure legend, the reader is referred to the web version of this article.)

To reveal the reason for this change, we compared the interacting structures between 3SX and the residues, Glu326 and Arg364, at 0 and 300 ns of the MD simulations. At 0 ns (Fig. 10a), the oxygen atom of 3SX is oriented toward the Glu326, and the hydrogen atoms of the terminal methyl group of 3SX face toward the hydrogen atoms of Arg364. Owing to the repulsive interactions between the hydrogen atoms of 3SX and those of Arg364, there is no attractive interaction between 3SX and Arg364. On the other hand, at 300 ns (Fig. 10b), the oxygen atom of 3SX is oriented toward the Arg364 and the hydrogen atoms face away from Arg364. As a result, strong attractive interactions between the oxygen atom and the hydrogen atoms of Arg364 form, leading to the strong peak in interaction energy between 3SX and Arg364 (Fig. 9). Consequently, the present MD simulations reveal that the attractive interaction between 3SX and Arg364 is significantly enhanced by the rotation of the terminal group in 3SX.

As for the interaction between 3SX and Glu326, a hydrogen atom of 3SX forms a weak interaction with Glu326 at 0 ns (Fig. 10a), whereas the three hydrogen atoms of 3SX interact strongly with an oxygen atom of Glu326 at 300 ns (Fig. 10b). Accordingly, the attractive interaction between 3SX and Glu326 is enhanced at 300 ns.

**Fig. 11:**
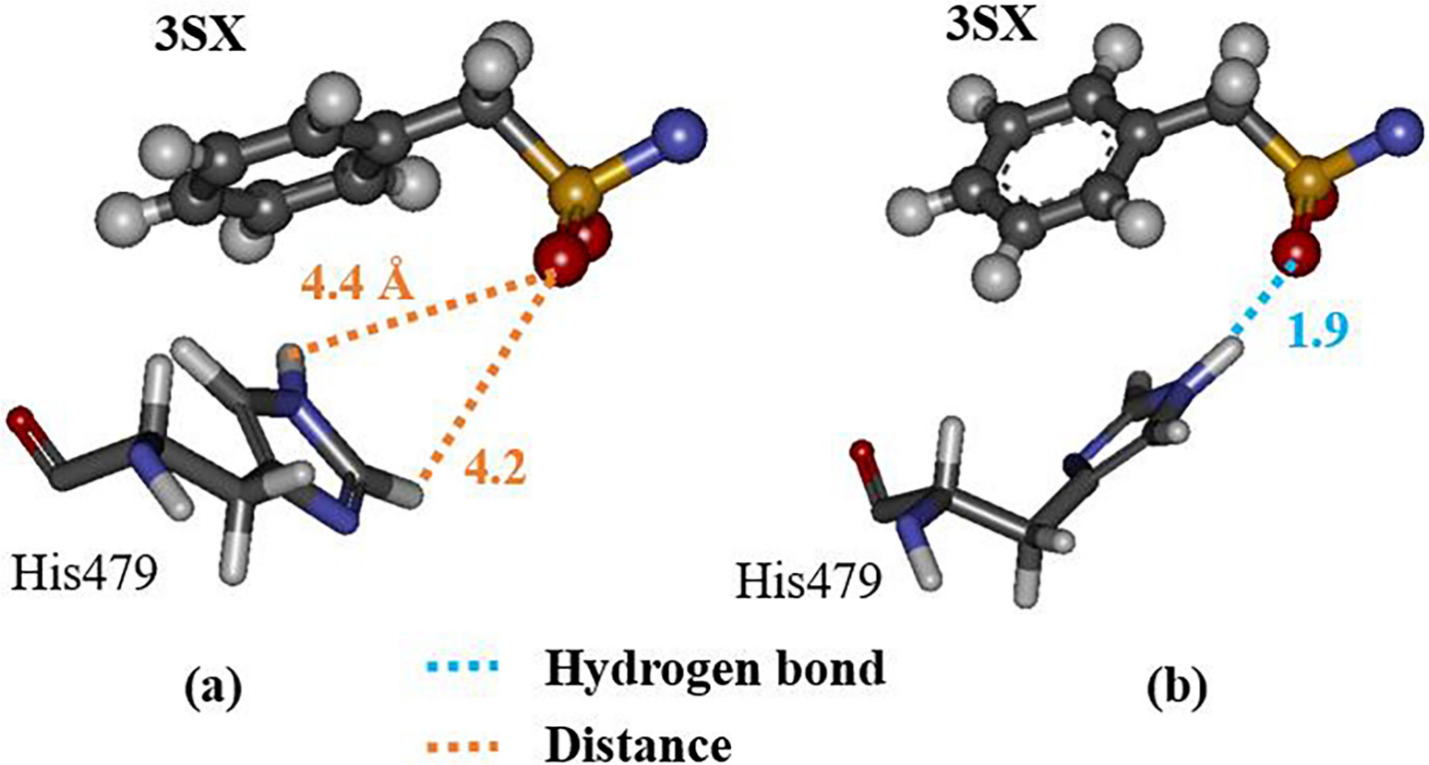
Change in interacting structure between 3SX and His479 in RORγt; (a) at 0 ns and (b) at 300 ns; only an interacting part of 3SX with His479 is shown.

As mentioned above, the interactions between 3SX and the residues, Arg364 and Glu326, in RORγt + 3SX are significantly enhanced by the structural change during the MD simulations. However, these residues do not interact directly with the residues of H12, so it is not likely that they contribute to the stability of H12. We therefore investigated the interaction between 3SX and His479, because the interaction energy between His479 and 3SX is significantly increased (Fig. 9). The attractive interaction energy increases by 13.8 kcal/mol in the MD simulation. His479 forms a strong hydrogen bond with the oxygen atom of 3SX at 300 ns (Fig. 11b), although they separated by more than 4 Å in the structure at 0 ns (Fig. 11a). Fig. 11 indicates that the rotation of the imidazole ring of His479 causes an enhancement of the interaction between His479 and 3SX.

His479 is located in H11 and is considered to contribute to the stabilization of H12 by the binding with Tyr502 located in H12 [15]. As shown in Fig. 8, Fig. 11, in the initial structure, His479 interacts with Tyr502 and there is no interaction with 3SX. However, at the 300 ns structure, His479 forms a strong hydrogen bond with 3SX and is thus separated from Tyr502. Accordingly, the present MD simulations elucidate that the conformational change of the His479 side chain induces a significant change in the interactions between His479, Tyr502, and 3SX, furthermore, this change is a trigger for a large change in the H12 conformation in RORγt + 3SX. Notably, such a change does not occur in the agonist-bound RORγt + 3SN.

**Fig. 12:**
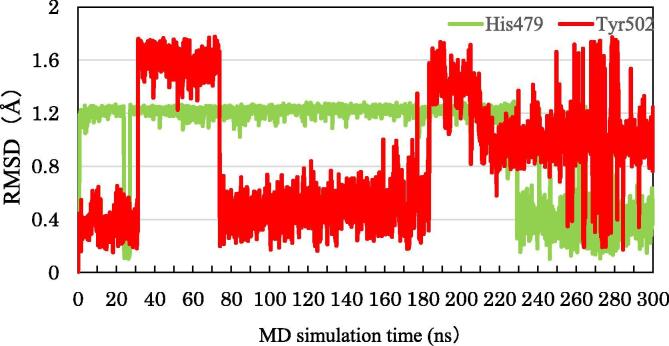
Change in RMSD of His479 and Tyr502 in RORγt + 3SX during MD simulations.

**Fig. 13:**
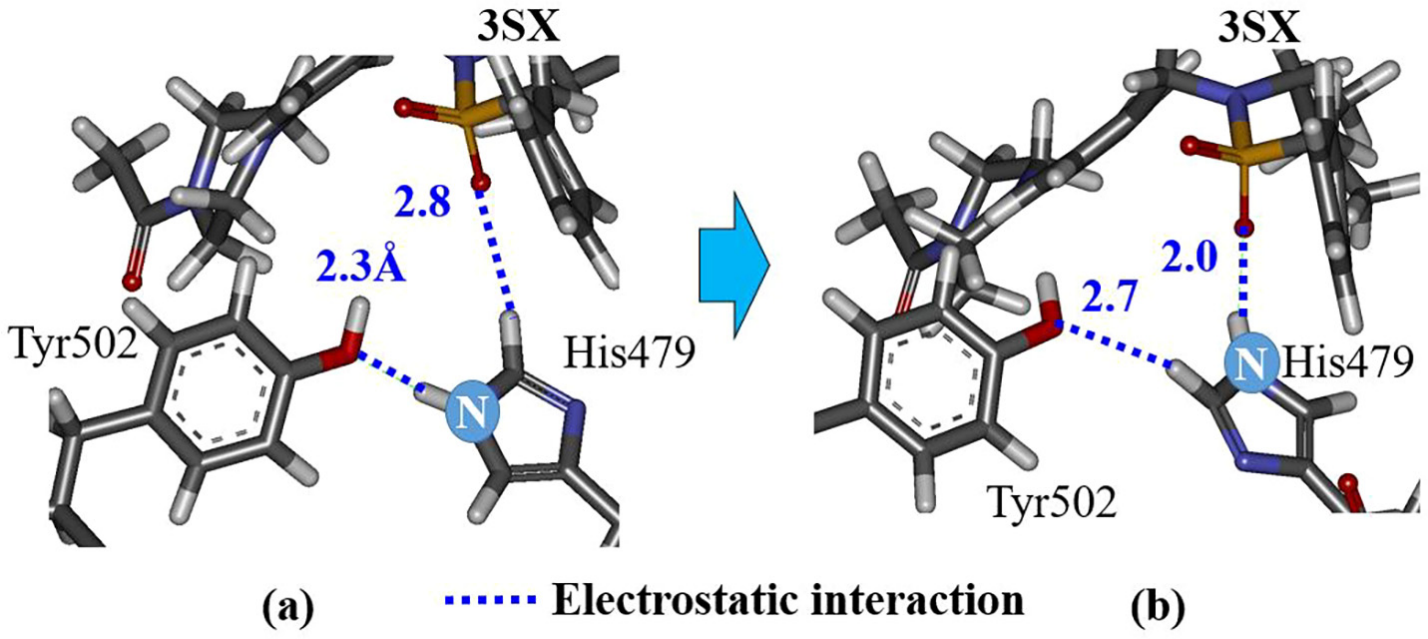
Conformational change of His479 side chain in RORγt + 3SX; (a) at 228 ns and (b) at 230 ns of MD simulations.

**Fig. 14:**
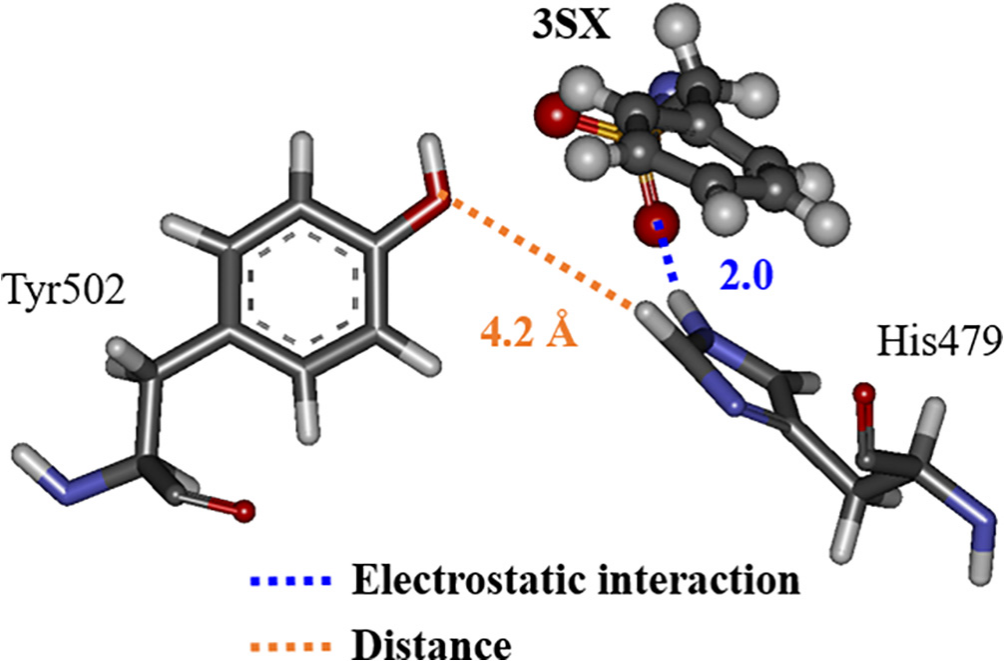
Interacting structure between 3SX, His479 and Tyr502 in RORγt + 3SX at 235 ns.

Moreover, we analyzed the change in the RMSDs of His479 and Tyr502, in order to elucidate the change in the interactions between them. Fig. 12 indicates that the structure of His479 changes drastically at 230 ns, whereas there are only small changes in the structure of Tyr502, however, after 240 ns it significantly fluctuates. It is likely that the change in the structure of His479 causes a weakening in the interaction between His479 and Tyr502 and triggers the fluctuations in the structure of Tyr502. We therefore analyzed the interacting structures of His479, Tyr502, and 3SX at around 230 ns. At 228 ns, the NH group of the imidazole ring of His479 interacts strongly with the side chain of Tyr502, while the CH group interacts weakly with 3SX (Fig. 13a). At 230 ns, the imidazole ring of His479 rotates 180° and the positions of NH and CH groups are exchanged (Fig. 13b). As a result, the distance between the NH group and the 3SX is 2.0 Å, and they interact strongly, whereas the CH group interacts weakly with the Tyr502 side chain. It is therefore elucidated that the rotation of the imidazole ring of His479 is the main reason for the weakening of the interaction between His479 and Tyr502, which leads to the fluctuation of H12. We furthermore confirmed that the separation between His479 and Tyr502 is increased at 235 ns, as shown in Fig. 14.

### 3.4 Change in interactions between agonist 3SN and RORγt residues

**Fig. 15:**
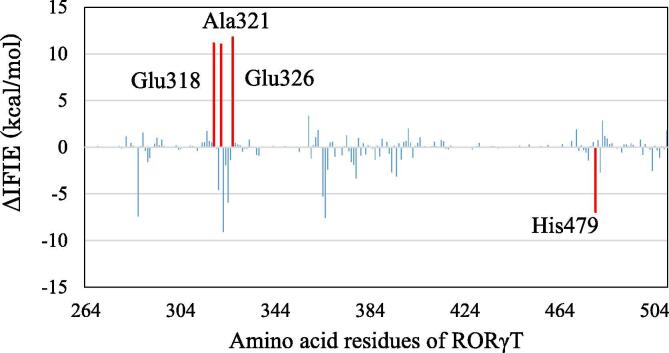
Difference in interaction energies (IFIE) between 3SN and each RORγt residue for the structures at 0 and 300 ns; red bars indicate the residues with difference in interaction energies larger than 10 kcal/mol. His479 is also marked by red. (For interpretation of the references to colour in this figure legend, the reader is referred to the web version of this article.)

**Fig. 16:**
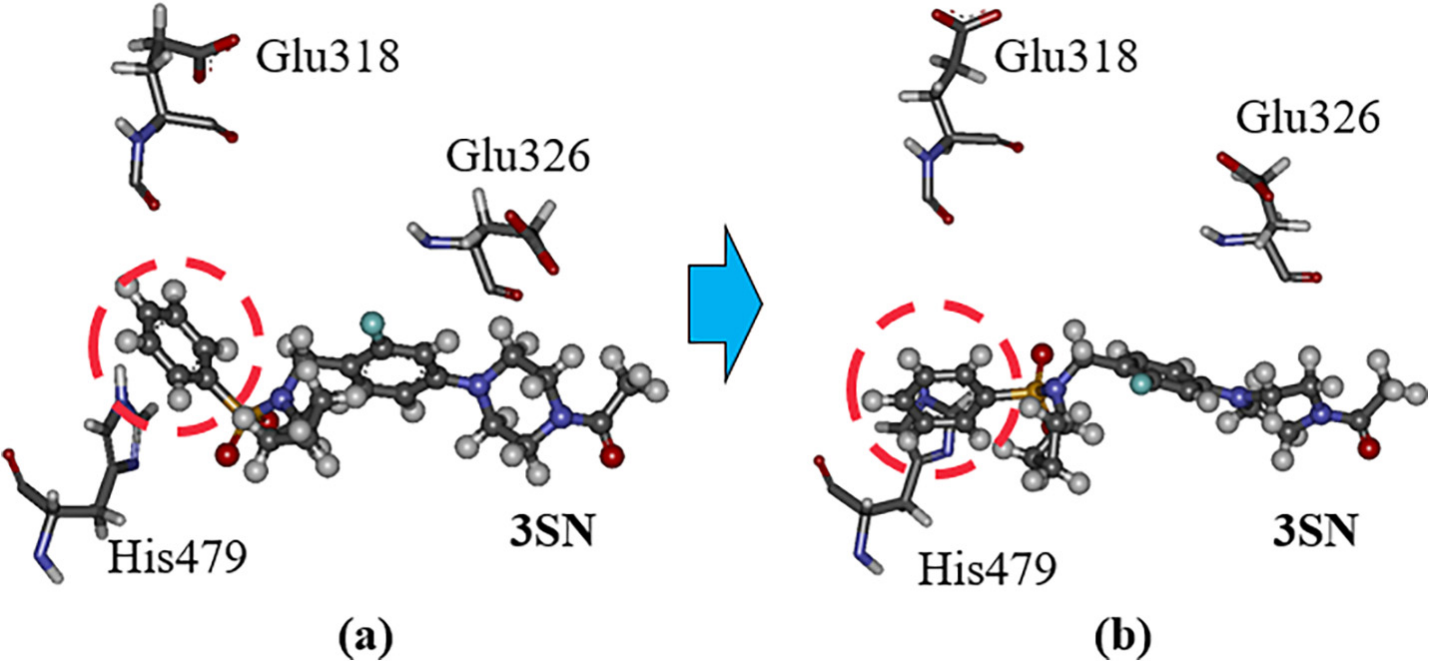
Change in interacting structure between 3SN, His479, Glu326 and Glu318 in RORγt; (a) at 0 ns and (b) at 300 ns.

To elucidate the difference in the effects of agonist and inverse-agonist bindings to RORγt, we investigated the change in the interactions between the RORγt residues for RORγt + 3SN. The change in interaction energies (IFIE) between 3SN and each RORγt residue is shown in Fig. 15. Compared with the result for the inverse-agonist-bound RORγt + 3SX (Fig. 9), the change in interaction energy is little. This result is reasonable, as the structural change of RORγt + 3SN is smaller than that of RORγt + 3SX. The interaction energies between 3SN and the residues, Glu318, Ala321, and Glu326 are significantly decreased during the MD simulations, while the interaction energy between 3SN and His479 is slightly increased by the structural change in RORγt + 3SN. To reveal the reason for these changes in the interaction energies, we analyzed the change in interacting structures between 3SN and these residues. As shown in Fig. 16, the conformation of the phenyl ring marked by a red circle of 3SN is significantly changed during the MD simulations. At 300 ns, the ring gets closer to His479, resulting in a stronger interaction between 3SN and His479. Notably, the enhancement in energy is only 6.9 kcal/mol, which is half as much as that for RORγt + 3SX. In contrast, Glu318 and Glu326 are further apart from 3SN at 300 ns, and their interactions with 3SN become weak.

**Fig. 17:**
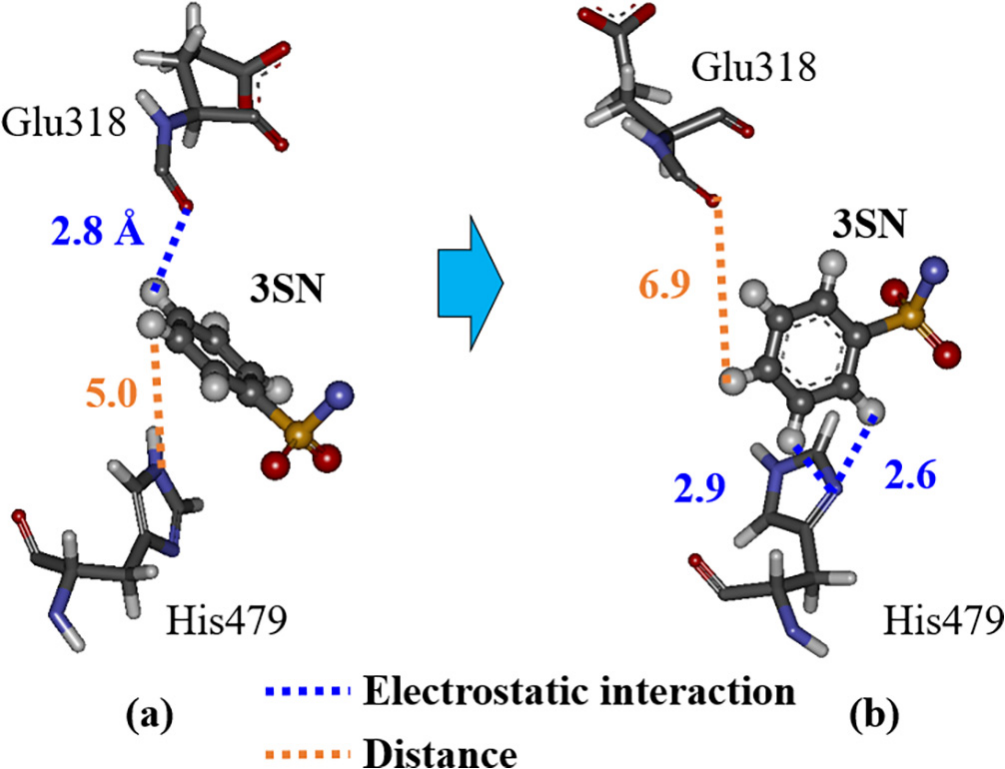
Change in interacting structures between 3SN, His479 and Glu318 in RORγt; (a) at 0 ns and (b) at 300 ns. Only an part around phenyl ring of 3SX is shown.

In the same way as for RORγt + 3SX, we checked in detail the interactions between the imidazole ring of His479 and the phenyl ring of 3SN. As shown in Fig. 17, both rings rotate significantly to produce two attractive electrostatic interactions between the nitrogen atom of the imidazole ring and the two hydrogen atoms of the phenyl ring. As a result, 3SN shifts to His479 and is separated from Glu318 as shown in Fig. 17b. This structural change is qualitatively consistent with the change in interaction energies shown in Fig. 15.

### 3.5 Comparison of ligand-binding effects on RORγt structure

The present MD simulations revealed that the H12 conformation of RORγt is drastically changed by the binding of the inverse agonist 3SX, while the binding of the agonist 3SN has no significant effect on the H12 conformation. The reason for this difference was also revealed that the interaction between the His479 in H11 and Tyr502 in H12 is weakened by the 3SX binding. This corresponds to the previous MD simulations [15]. In addition, the present MD simulations found that the rotation of the imidazole ring in His479 induced by 3SX is closely related with the weakening of the interaction between His479 and Tyr502. However, it is not evident why 3SN and 3SX have such different influences on the RORγt structure, although their chemical structures are similar each other as shown in Fig. 1. Our results elucidate on this and we will now discuss it in further detail.

**Fig. 18:**
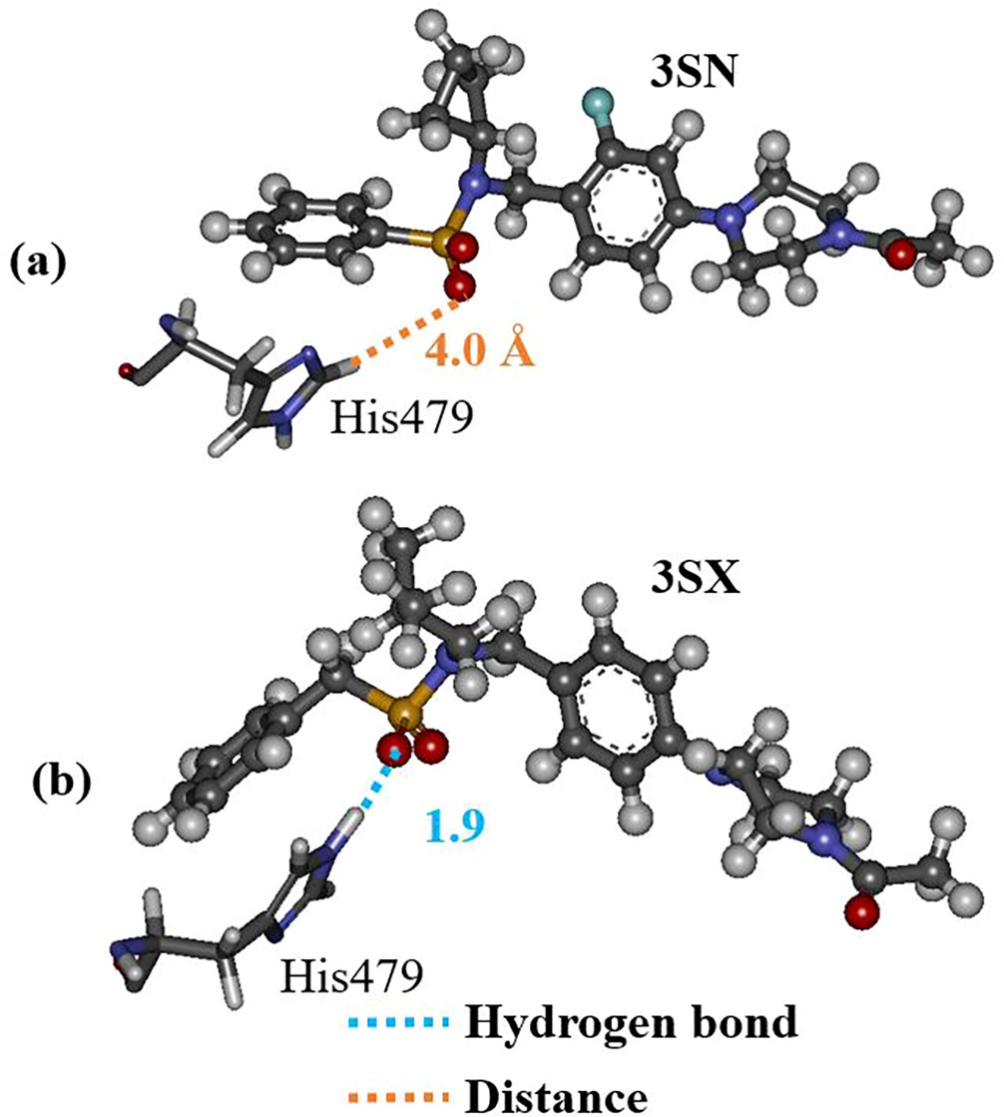
Interactions between His479 and (a) 3SN or (b) 3SX in the structure at 300 ns.

It can be considered that the rotation of the imidazole ring of His479 is induced by the interaction between the ring and the oxygen atom in 3SX. In fact, Fig. 13a clearly indicates that the distance between the ring and the oxygen atom is 2.8 Å in the structure at 228 ns, therefore the oxygen atom of 3SX can interact with the other atoms of the ring. In addition, in the structure at 300 ns (Fig. 18b), the oxygen atom forms a strong hydrogen bond with the imidazole ring, leading to a significant change in the conformation of His479. On the other hand, the oxygen atom of 3SN is separated by 4 Å from the imidazole ring at 300 ns, as shown in Fig. 18a. Therefore, there is no strong interaction between 3SN and His479, and the interaction between His479 and Tyr502 remains intact.

**Fig. 19:**
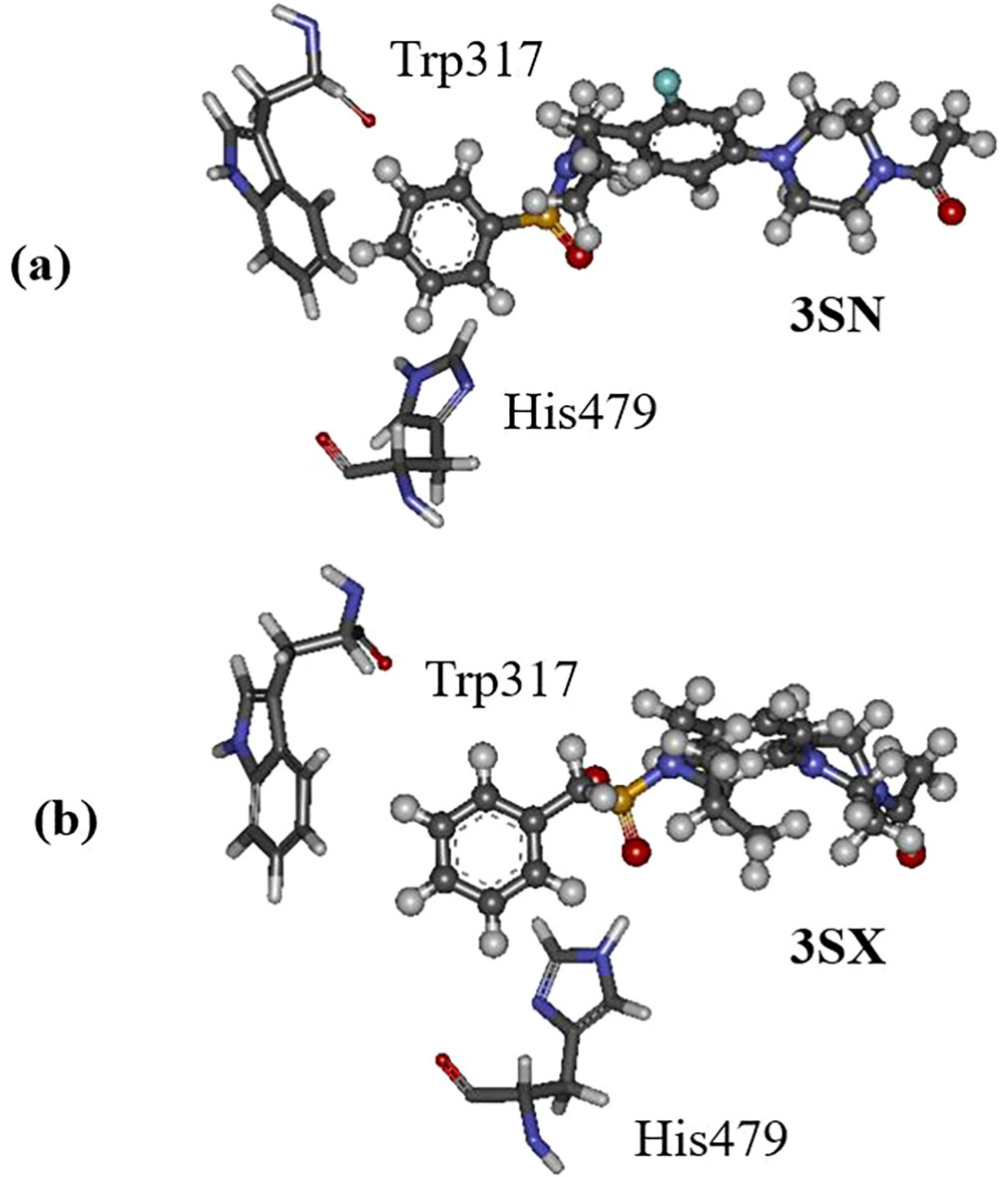
Relative positions of His479, Trp317 and ligand at 300 ns; (a) 3SN and (b) 3SX.

It is expected that the distance between His479 and 3SN/3SX is related to the conformation of Trp317, because Trp317 is located near both His479 and the ligands. In the previous MD simulations [15], the conformational change of Trp317 was found to be related to the functions of the agonist and inverse agonist for RORγt. We thus analyzed the relative positions of His479, Trp317 and the ligand in the structure at 300 ns. As shown in Fig. 19, the indole ring of Trp317 exists on the left side of the phenyl ring of the ligand. Because of the steric hindrance between these rings in RORγt + 3SN, 3SN cannot get closer to His479, as shown in Fig. 19a. On the other hand, in RORγt + 3SX, there is empty space around the phenyl ring of 3SX, and it can move to the imidazole ring of His479, resulting in the strong hydrogen bond between the oxygen atom of 3SX and the imidazole ring (Fig. 18a). It is expected that a ligand, which can bind to His479 and induce the rotation of its imidazole ring, can be a potent inverse agonist for RORγt.

Finally, we will discuss the reason why the slight difference in the structures of 3SN and 3SX cause a significant difference in their influence on the structure of RORγt. As marked by a red circle in Fig. 1, the distance between the sulfur atom and the phenyl ring is different in 3SN and 3SX. The longer bond in 3SX means its terminal phenyl ring can reach further into the ligand-binding pocket of RORγt without any steric hindrance. As a result, the oxygen atom of 3SX can get closer to the imidazole ring in His479 to form a hydrogen bond (Fig. 18b). On the other hand, the short bond in 3SN results in steric hindrance to the RORγt residues existing around the ligand-binding pocket, and 3SN cannot reach as deeply into the pocket. As a result, the oxygen atom in 3SN cannot get close to the imidazole ring in His479 (Fig. 19a). Consequently, it can be concluded that a slight difference in the 3SN and 3SX structures around the sulfur atom induces the different influences on the conformations of Trp317 and His479, which are important for the stability of H12 in RORγt.

## 4 Conclusions

To determine how the binding of an agonist or an inverse agonist effects the structure of retinoic acid receptor-related orphan receptor gamma (RORγt), we used MD simulations to investigate structural changes. The electronic states for certain characteristic structures obtained were further analyzed by *ab initio* FMO calculations to elucidate the essential interactions between the amino acid residues of RORγt, in order to distinguish the effects of the agonist and the inverse-agonist bindings. The conformation of H12 in RORγt was found to key in explaining the different function of the agonist and the inverse agonist on RORγt. On the binding of the agonist to RORγt, the conformation of H12 does not change, owing to the specific hydrogen bond between His479 of H11 and Tyr502 of H12. In contrast, the H12 conformation in the inverse-agonist-bound RORγt changes significantly to separate from the H11 structure. These results can explain the difference in the functions of the agonist and the inverse agonist on RORγt. Furthermore, when the inverse agonist binds to RORγt, the imidazole ring of His479 rotates, significantly weakening the interaction between His479 and Tyr502. As a result, H12 is separated from the other helixes within RORγt. We conclude that this conformational change of His479 in RORγt is a main reason for the H12 destabilization in the inverse-agonist-bound RORγt. We are currently underway with the MD simulations for other complexes of RORγt with different agonist/inverse-agonist, in order to confirm that our present finding is applicable to the other ligands.

## Author statement

There is no statement from authors.

## Declaration of Competing Interest

The authors declare that they have no known competing financial interests or personal relationships that could have appeared to influence the work reported in this paper.
