# Supplementary material for: Structural change of retinoic-acid receptor-related orphan receptor induced by binding of inverse-agonist: Molecular dynamics and ab initio molecular orbital simulations
Source: Comput Struct Biotechnol J. 2020 Jun 25;18:1676–85. doi: 10.1016/j.csbj.2020.06.034 (PMC7338990; doi:10.1016/j.csbj.2020.06.034)

**Supplementary data**

Figure S1 Modeling of initial structures of RORγT with 3SX having H11’ and H12 domains


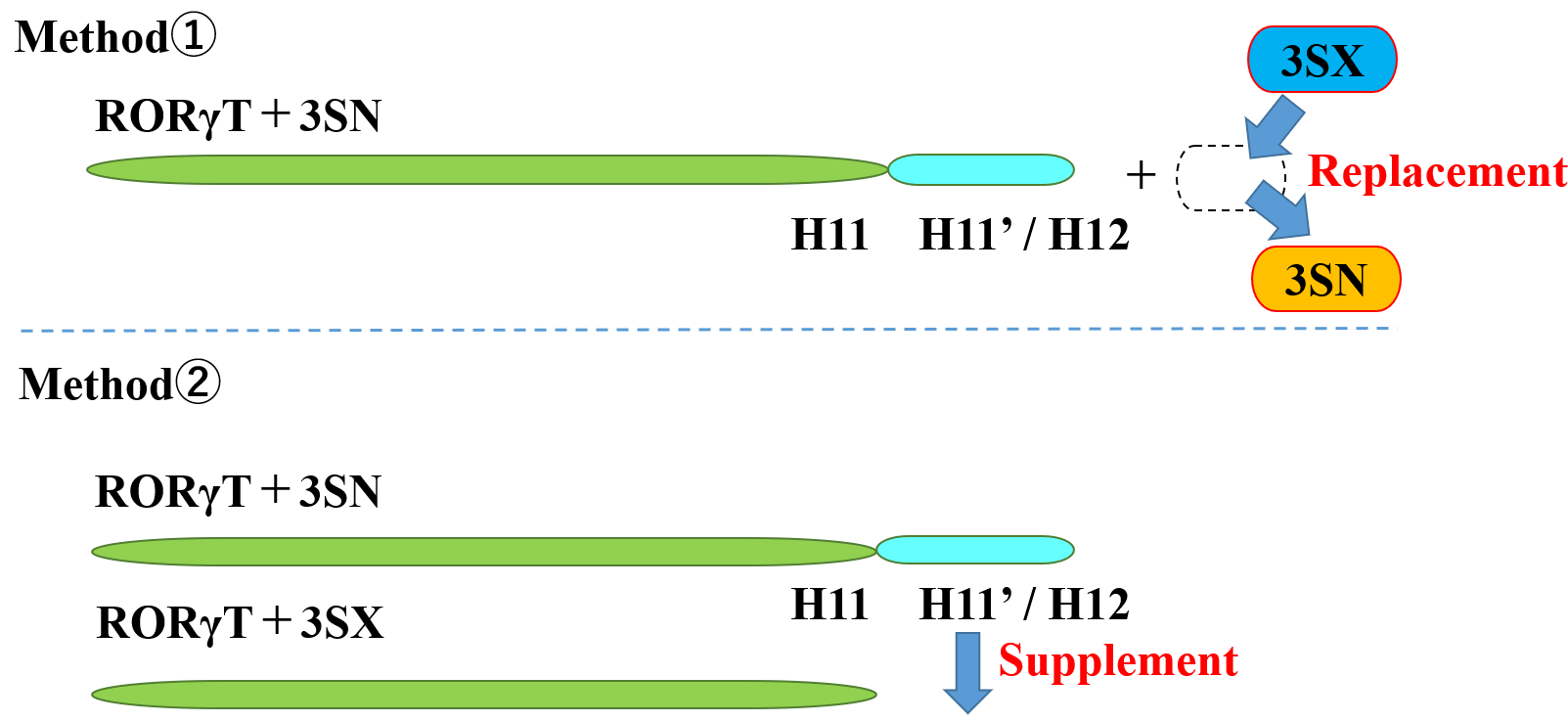


Figure S2 Protonation states of His residue


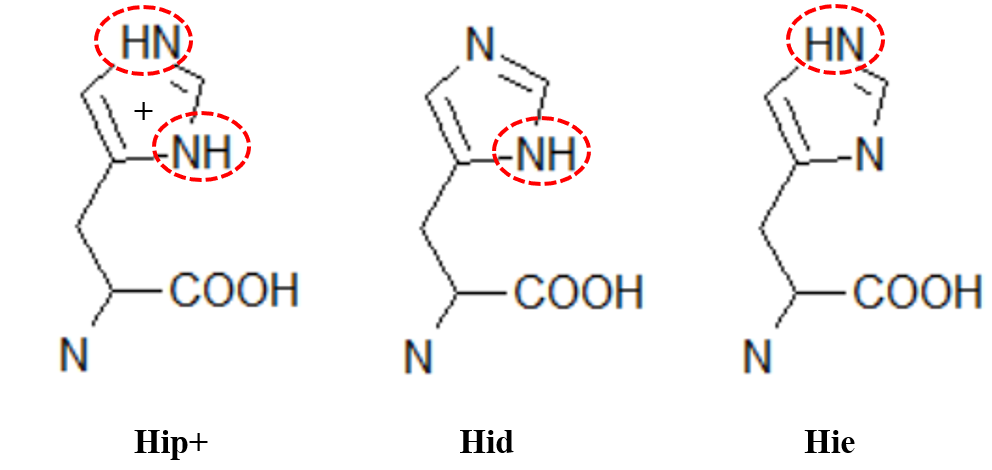

Supplement: Supplementary data 1 [file mmc1.docx]
